# Identification of signalling cascades involved in red blood cell shrinkage and vesiculation

**DOI:** 10.1042/BSR20150019

**Published:** 2015-04-16

**Authors:** Elena B. Kostova, Boukje M. Beuger, Thomas R.L. Klei, Pasi Halonen, Cor Lieftink, Roderick Beijersbergen, Timo K. van den Berg, Robin van Bruggen

**Affiliations:** *Department of Blood Cell Research, Sanquin Research, Plesmanlaan 125, 1066CX, Amsterdam, The Netherlands; †Division of Molecular Carcinogenesis, NKI Robotics and Screening Center, Plesmanlaan 121, 1066CX, Amsterdam, The Netherlands

**Keywords:** bioactive small molecule, compound screen, kinase inhibitor, red blood cell, vesiculation, AMPK, AMP-activated kinase, ATA, aurintricarboxylic acid, BCR-ABL, breakpoint cluster region protein–Abelson murine leukaemia viral oncogene homologue 1, CaM, calmodulin, CK2, casein kinase 2, Epo, erythropoietin, ERK, extracellular signal-regulated kinase, GPCR, G protein-coupled receptor, Jak, Janus kinase, LOPAC, Library of Pharmacologically Active Compounds, MAPK, mitogen-activated protein kinase, MEK, mitogen-activated protein kinase kinase, NO, nitric oxide, nRTK, non-receptor tyrosine kinase, PC, phosphatidylcholine, PDGFR, platelet-derived growth factor receptor, PI3K, phosphoinositide 3-kinase, PKC, protein kinase C, PLC, phospholipase C, PS, phosphatidylserine, RBC, red blood cell, RTK, receptor tyrosine kinase, SAGM, saline-adenine-glucose-mannitol, SCD, sickle cell disease, SMase, acid sphingomyelinase, STAT, signal transducer and activator of transcription, VEGFR, vascular endothelial growth factor receptor, β-AR, β-adrenergic receptor

## Abstract

Even though red blood cell (RBC) vesiculation is a well-documented phenomenon, notably in the context of RBC aging and blood transfusion, the exact signalling pathways and kinases involved in this process remain largely unknown. We have established a screening method for RBC vesicle shedding using the Ca^2+^ ionophore ionomycin which is a rapid and efficient method to promote vesiculation. In order to identify novel pathways stimulating vesiculation in RBC, we screened two libraries: the Library of Pharmacologically Active Compounds (LOPAC) and the Selleckchem Kinase Inhibitor Library for their effects on RBC from healthy donors. We investigated compounds triggering vesiculation and compounds inhibiting vesiculation induced by ionomycin. We identified 12 LOPAC compounds, nine kinase inhibitors and one kinase activator which induced RBC shrinkage and vesiculation. Thus, we discovered several novel pathways involved in vesiculation including G protein-coupled receptor (GPCR) signalling, the phosphoinositide 3-kinase (PI3K)–Akt (protein kinase B) pathway, the Jak–STAT (Janus kinase–signal transducer and activator of transcription) pathway and the Raf–MEK (mitogen-activated protein kinase kinase)–ERK (extracellular signal-regulated kinase) pathway. Moreover, we demonstrated a link between casein kinase 2 (CK2) and RBC shrinkage via regulation of the Gardos channel activity. In addition, our data showed that inhibition of several kinases with unknown functions in mature RBC, including Alk (anaplastic lymphoma kinase) kinase and vascular endothelial growth factor receptor 2 (VEGFR-2), induced RBC shrinkage and vesiculation.

## Abstract 2

After screening two libraries of small bioactive molecules and kinase inhibitors, we identified several signalling pathways to be involved in red blood cell (RBC) shrinkage and vesiculation. These include the Jak (Janus kinase)–STAT (signal transducer and activator of transcription) pathway, phosphoinositide 3-kinase (PI3K)–Akt pathway, the Raf–MEK (mitogen-activated protein kinase kinase)–ERK (extracellular signal-regulated kinase) pathway and GPCR (G protein-coupled receptor) signalling.

## INTRODUCTION

Transfusion of red blood cells (RBCs) is often a life-saving therapy for which no substitute exists. RBCs are transfused to improve oxygen supply in a diverse group of patients, including patients that suffer from trauma, haemoglobinopathies, cancer, surgical procedures or sepsis. Although the beneficial effects of transfusing RBC are clear, the use of RBC can also have adverse effects. RBC undergo considerable changes during storage, including lactate accumulation, decrease in ATP and 2,3-DPG (2,3-diphosphoglycerate) levels [1] and vesicle release [2]. These modifications, known as the storage lesion, lead to alterations in the function and lifespan of RBC *in vivo* post transfusion [3,4], which can have detrimental side effects in the recipient [5]. We and others have shown that after transfusion stored RBC release phosphatidylserine positive (PS^+^) vesicles which support the coagulation cascade [6–8] and can scavenge nitric oxide (NO) [9–11], leading to thrombosis and vasoconstriction in the recipient respectively. In addition, RBC vesicle shedding has been implicated in immunomodulation [12]. Vesicles released during storage induce the production of pro-inflammatory cytokines by monocytes promoting T-cell proliferation [12]. Moreover, generation of inflammatory vesicles is observed in sickle cell disease (SCD) via activation of acid sphingomyelinase (SMase) followed by ceramide accumulation [13]. The vesicles that are released are subsequently engulfed by monocytes promoting the production of pro-inflammatory cytokines and endothelial cell adhesion [13]. Furthermore, several bioactive lipids are downstream of SMase and ceramide, including PS and ceramide production, has been linked to PS exposure and cell shrinkage in RBC [14].

As mentioned, we have previously demonstrated that stored RBC spontaneously shed PS^+^ vesicles in an *in vitro* transfusion model [6]. Phospholipid membrane asymmetry is regulated by three enzymes: flippase, floppase and scramblase [15]. The flippase, also called aminophospholipid translocase, is an ATP-dependent inward-directed enzyme which transports lipids including PS and phosphatidylethanolamine (PE) to the inner leaflet of the plasma membrane [16], whereas the floppase, also known as multidrug resistant protein 1, is an outward-directed enzyme responsible for keeping phosphatidylcholine (PC) on the outside of the cell membrane [17]. The scramblase, on the other hand, is able to transport lipids across the membrane in a bidirectional manner [18]. Recently, increasing evidence in literature has demonstrated that the ion channel Tmem16f (transmembrane protein 16F) also functions as the calcium-activated scramblase [19–21]. During RBC storage, flippase activity is strongly reduced due to ATP depletion and potassium leakage [6]. Moreover, increased scramblase activity is observed due to elevated intracellular calcium levels. These events collectively lead to loss of membrane asymmetry, exposure of PS on the cell surface and finally vesicle shedding [6].

Vesiculation is not only relevant in the context of RBC storage and transfusion, but is also important during RBC aging and clearance *in vivo* [4]. Under physiological conditions, RBC has a lifespan of 120 days, which implies that 0.8% of total RBC are cleared per day. Furthermore, RBC becomes smaller and denser with age, a process facilitated by the release of vesicles containing haemoglobin [22,23]. Loss of membrane results in less deformable RBC which can no longer pass through the endothelial slits ultimately leading to their phagocytosis by red pulp spleen macrophages lining the endothelium [22,24]. All these data suggest that RBC vesiculation is beneficial when taking place in the spleen as a clearance mechanism [25], but deleterious when occurring in circulation after transfusion [5,6,9].

Even though RBC vesicle release is a well-documented phenomenon, little is known about the exact signalling pathways that underlie this process. In the present study, we aimed at identifying signalling cascades involved in RBC vesiculation by screening the effect of compounds from two different libraries of bioactive small molecules on RBC vesicle shedding and shrinkage. Using these two libraries, the library of pharmacologically active compounds (LOPAC) and the Selleckchem Kinase Inhibitor Library, we confirmed the importance of well-known pathways such as calcium signalling [26], caspase activity [27] and PKC (protein kinase C) signalling [28], but we also discovered several cascades not described previously to play a role in RBC vesiculation. These include G protein-coupled receptor (GPCR) signalling via antagonism of β-adrenergic (β-AR) and P2Y receptors, the phosphoinositide 3-kinase (PI3K)–Akt (protein kinase B) pathway, the Jak (Janus kinase)–STAT (signal transducer and activator of transcription) pathway and the Raf–MEK (mitogen-activated protein kinase kinase)–ERK (extracellular signal-regulated kinase) pathway. Moreover, we propose a novel role for casein kinase 2 (CK2) in RBC shrinkage through modulation of the Gardos channel via calmodulin (CaM). In addition, our data suggest that anaplastic lymphoma kinase (Alk) kinase and vascular endothelial growth factor receptor 2 (VEGFR-2) are involved in the regulation of RBC shrinkage and vesiculation.

## MATERIALS AND METHODS

### Reagents

The LOPAC library, the calcium ionophore ionomycin A23187, TRAM-34 and buffer reagents were provided by Sigma (Sigma–Aldrich). The Kinase Inhibitor Library, containing 192 chemicals, was obtained from Selleckchem (Selleckchem).

### Red blood cell isolation

Heparinized venous blood was obtained from healthy volunteers after informed consent. Blood studies were approved by the Medical Ethical Committee of Sanquin Research and performed in accordance with the 2013 Declaration of Helsinki. RBCs were isolated in the following manner: blood was centrifuged at 210 ***g* at** room temperature for 15 min. Next, plasma and buffy coat were removed and RBC were washed twice with saline–adenine–glucose–mannitol medium (SAGM medium, 150 mM NaCl, 1.25 mM adenine, 50 mM glucose, 29 mM mannitol, pH 5.6; Fresenius SE) for 5 min at 570 ***g***. Finally, RBCs were resuspended in SAGM medium and counted on an ADVIA 2120 Hematology System (Bayer Healthcare AG).

### LOPAC and Kinase Inhibitor Library screening

Freshly isolated RBCs were washed in HEPES buffer (132 mM NaCl, 20 mM HEPES, 6 mM KCl, 1mM MgSO_4_, 1.2mM K_2_HPO_4_) supplemented with 2 mM CaCl_2_ and 10 mM glucose. RBCs (0.5 × 10^6^) were plated per well in duplo on a flat-bottom 96-well plate pre-coated with compounds from the LOPAC or the Kinase Inhibitor Library at 10 μM concentration, for all primary screens and incubated at 37°C for 30 min. Next, RBCs were treated with HEPES or with 5 μM ionomycin at 37°C for 30 min. Validation screens were performed analogously, with compound concentrations: 100 nM, 1 μM and 10 μM. All screens were performed with three different donors, besides LOPAC primary screens which were performed with two donors. All screens were run on a LSRII + HTS (BD Bioscsiences) flow cytometer. Raw data were analysed with FACSDiva Software (BD Biosciences).

### Library screening hit selection and statistics

Separate screens were done with the LOPAC and the Kinase Inhibitor Library and for both libraries there were different screens for inhibition and induction. In all four cases, a primary and a validation screen were performed, making a total of eight screens. In most screens raw data were plate-normalized via dividing by the median of the negative controls on the plate. The only exception was the primary kinase inhibitor screen where the plate normalization was done via dividing per median of the plate, due to problems with the negative controls. In case of the primary screens, a z-score calculation was done per replicate and readout (P1, P2, P3). Over the replicates and per readout the mean of the z-scores was calculated. The mean z-score for the most interesting readout (P2) was used as a selection criterion. In general, in case of induction screens, the 30 highest values were selected for validation and in case of inhibition screens the lowest 30. In the validation screens three different concentrations of the drugs were used. Per compound and concentration the median value over three replicates was calculated. Furthermore a *t* test was performed comparing the values for the three replicates with a NULL distribution consisting of the negative controls. The resulting *P*-values were corrected for multiple testing using the Benjamin–Hochberg method [29]. Adjusted *P*-value (Padj score) of ≤0.1 was considered significant. Most compounds were considered validated hits when their median of the replicates (Rep Median score) was smaller than 1, in case of inhibition screens and higher than 1 in case of induction screens and the adjusted *P*-value (Padj score) was smaller than 0.1 with the exception of eight compounds which were validated according to cell scatter. All calculations were performed using programming language R. For plate normalization and z-scoring R package cell HTS2 version 2.8.3 [30] was used.

## RESULTS

### Set-up and analysis of the LOPAC and the Kinase Inhibitor primary library screens

**Figure 1:**
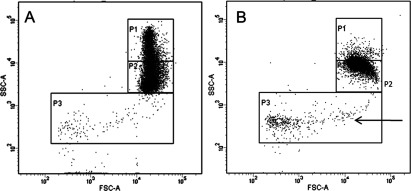
Scatter plots and gating strategy of RBC with DMSO (A) and with (B) ionomycin stimulation In a control setting with DMSO treatment alone (**A**) RBC demonstrate 2500 events in P1, 7500 events in P2 and 200 events in P3. Upon stimulation with 5 μM ionomycin at 37°C for 30 min (**B**), RBC shrink due to Ca^2+^ flux and K^+^ efflux, leading to vesiculation (arrow) accompanied by an increase in events in P2 to 9000. Plots represent one of six independent measurements.

First, we performed four primary screens: LOPAC inhibition and induction screens and Kinase Inhibitors Library inhibition and induction screens. Each LOPAC screen contained 1280 small molecules with known biological functions in the fields of cell signalling and neuroscience, including apoptosis, gene regulation and expression, lipid signalling, neurotransmission, phosphorylation, ion channel transport and G-protein signalling. The Selleckchem Kinase Inhibitor Library consisted of 192 inhibitors specifically targeting kinases from various families. We performed inhibition and induction screens in parallel since we aimed at identifying molecules that induce RBC vesiculation (induction screens) and molecules that inhibit vesiculation induced by ionomycin (inhibition screens). In a control setting without stimulation (DMSO alone), RBC scatter could be depicted by three populations (P) each containing a specific number of events. P1 contained 2500 events; P2 contained 7500 events, whereas P3, the gate in which larger vesicles/microparticles could be observed, contained 200 events (Figure 1A). When RBCs were treated with ionomycin, a change in scatter accompanied by a considerable change in the number of events in all the three gates was observed. Events from P1 moved to P2, which reached over 9000 events, corresponding to a reduction in side scatter (cell shrinkage); furthermore around 500 events were detected in P3 (Figure 1B; arrow). However, in our analysis, we refer to RBC vesiculation as increase in events in gate P2. We chose this parameter and not the increase in P3, as the majority of vesicles will be too small to be detected on the flow cytometer and other events, such as debris, also fall into this gate. This renders P3 unsuitable for quantification of the effects of the different compounds. On the other hand, cell shrinkage due to membrane loss invariably occurs following vesiculation and can be depicted as an increase in the number of events in P2, accompanied by a decrease in events in P1. As a calcium ionophore, ionomycin induces calcium influx in RBC, which stimulates the calcium-activated potassium channel KCNN4 (potassium intermediate/small conductance calcium-activated channel, subfamily N, member 4) (IK-1, inwardly rectifying potassium current; SK4, small conductance calcium-activated potassium channel 4; Gardos channel) leading to potassium efflux, cell dehydration, cell shrinkage, PS exposure and ultimately vesicle release [31–33]. As inducer of vesiculation, ionomycin was used as a positive control in all induction screens. After analysis of the primary LOPAC screens we identified 123 compounds inducing vesiculation and 162 compounds inhibiting vesiculation, which accounted for roughly 10% of all tested compounds. However, of each set we selected the top 2.3% of all compounds, considering normal distribution, which accounted for the 29 hits that were chosen for further validation. After analysis of the primary kinase inhibitor screens, it became clear that the inhibition screen did not yield any hits. For the induction screen of the Kinase Inhibitor Library we selected hits for further validation based on z-score, which accounted for 21 compounds in total.

### Set-up and analysis of LOPAC and Kinase Inhibitor Library validation screens

**Figure 2:**
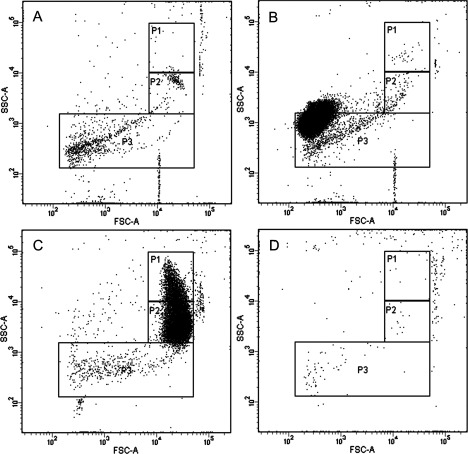
Scatter plots of RBC treated with LOPAC compounds palmitoyl-DL-carnitine chloride, calmidazolium chloride, SCH-202676 hydrobromide and rac-2-ethoxy-3-hexadecanamido-1-propylphosphocholine Palmitoyl-DL-carnitine chloride (PKC modulator; **A**) and calmidazolium chloride (CaM antagonist; **B**) induced vesiculation assessed by transfer of all events from P2 to P3. SCH-2022676 (GPCR modulator) caused vesiculation (increased number of events in P3) but no shrinkage (no change in P2; **C**), whereas rac-2-ethoxy-3-hexadecanamido-1-propylphosphocholine had a pronounced effect on RBC preventing the measurement of any events (**D**). RBCs were treated with the respective compounds for 30 min at 37°C, followed by flow cytometry. All compounds were diluted in DMSO and added at 10 μM final concentration. Plots represent one of three independent measurements.

**Figure 3:**
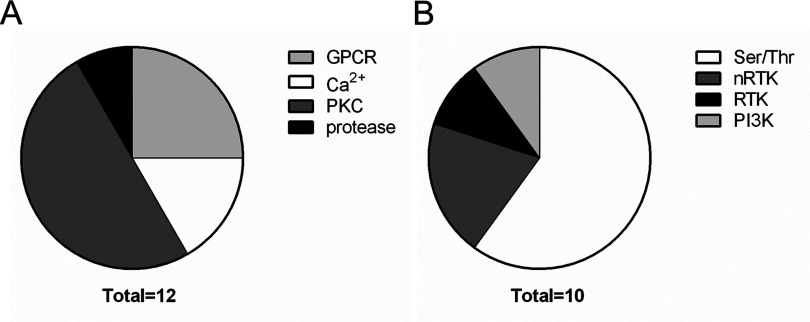
Validated compounds from LOPAC and Kinase Inhibitor Library induction screens clustered according to function We identified 12 LOPAC compounds inducing RBC shrinkage and vesiculation, which were divided in four functional groups (**A**): GPRC signalling (bromoacetyl alprenolol menthane, reactive Blue 2, SCH-202676 hydrobromide), Ca^2+^ signalling (calmidazolium chloride and NNC 55-0396), PKC related (ET-18-OCH3, rottlerin, PMA, tamoxifen citrate, palmitoyl-DL-carnitine chloride, rac-2-ethoxy-3-hexadecanamido-1-propylphosphocholine) and protease related (ATA). The validated compounds from the Kinase Inhibitor Library inducing RBC shrinkage and vesiculation were divided in four groups according to kinase targets (**B**): serine/threonine (A-769662, BIRB 796, sorafenib tosylate, SNS-314 mesylate, CX-4945, AT7867), nRTK (NVP-BSK805, AP24534), RTK (NVP-TAE684) and PI3K (AS-252425).

**Figure 4:**
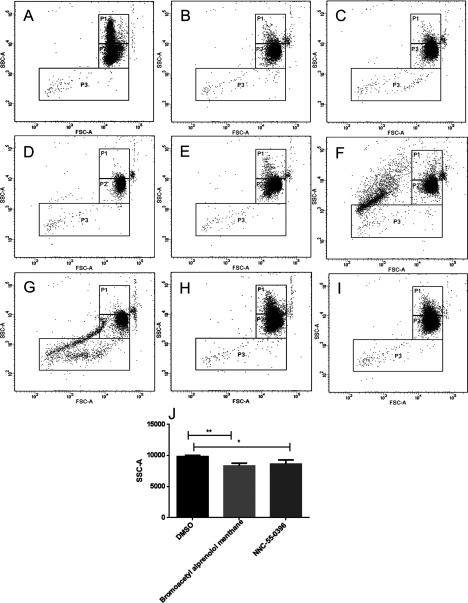
Validated compounds from the LOPAC library inducing RBC shrinkage and vesiculation RBCs were treated with DMSO control (**A**), ATA (calpain inhibitor; **B**), tamoxifen citrate (PKC inhibitor; **C**), reactive blue 2 (P2Y antagonist; **D**), ET-18-OCH3 (PKC inhibitor; **E**), rottlerin (PKC inhibitor; **F**), PMA (PKC activator; **G**), bromoacetyl alprenolol menthane (β-AR antagonist; **H**) and NNC 55-0396 (T-type Ca^2+^ channel blocker; **I**). RBC shrinkage and vesiculation were measured by the significant increase in events in P2, compared with DMSO control and was observed in all conditions, with the exception of bromoacetyl alprenolol menthane (**H**) and NNC 55-0396 (**I**) which induced RBC shrinkage measured by a significant decrease RBC side scatter (**J**). RBCs were treated with the respective compounds for 30 min at 37°C followed by flow cytometry. All compounds were diluted in DMSO and added at 10 μM final concentration. Plots represent one of three independent measurements. Quantification of cell side scatter (SSC-A) upon DMSO, bromoacetyl alprenolol menthane and NNC 55-0396 treatment (**J**); results shown represent mean±S.D., *n*=3; *P<0.1, **P<0.01, unpaired *t* test was applied during the analysis.

**Figure 5:**
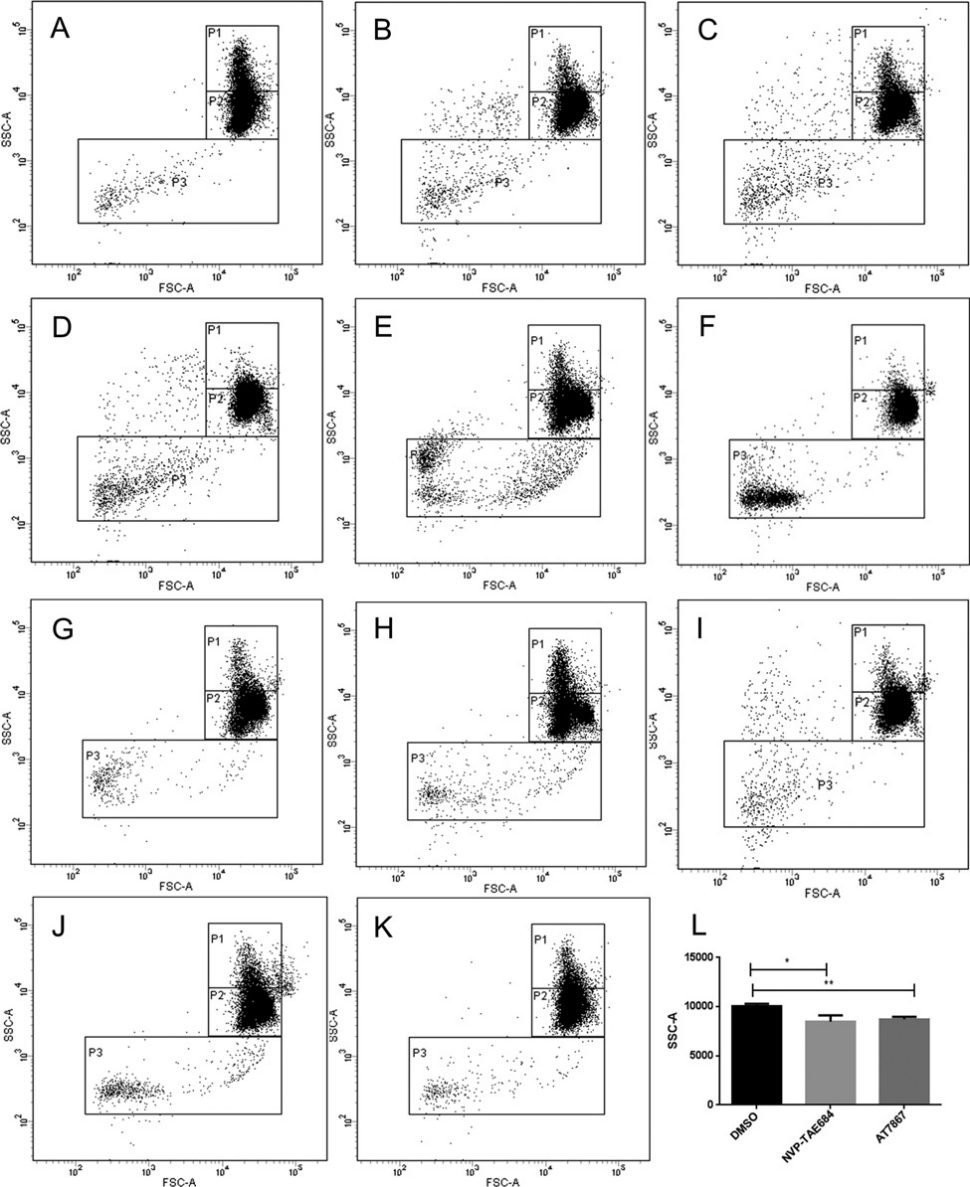
Validated compounds from the Kinase Inhibitor Library inducing RBC shrinkage and vesiculation RBCs were treated with DMSO alone as a control (**A**). CX-4945 (CK2 inhibitor; **B**), AS-25245 (PI3K inhibitor; **C**), NVP-BSK805 (Jak inhibitor; **D**), sorafenib tosylate (Raf-1 inhibitor; **E**), AP24534 (pan–BCR–ABL inhibitor; **F**), BIRB 796 (p38 MAPK inhibitor; **G**), SNS-314 mesylate (Aurora kinase inhibitor; **H**) and A-7699662 (AMPK activator; **I**), induced RBC shrinkage and vesiculation measured by an increase in events in P2, whereas NVP-TAE684 (Alk inhibitor; **J**) and AT7867 (Akt inhibitor; RBCs were treated with DMSO alone as a control (**A**). CX-4945 (CK2 inhibitor; **B**), AS-25245 (PI3K inhibitor; **C**), NVP-BSK805 (Jak inhibitor; **D**), sorafenib tosylate (Raf-1 inhibitor; **E**), AP24534 (pan–BCR–ABL inhibitor; **F**), BIRB 796 (p38 MAPK inhibitor; **G**), SNS-314 mesylate (Aurora kinase inhibitor; **H**) and A-7699662 (AMPK activator; **I**), induced RBC shrinkage and vesiculation measured by an increase in events in P2, whereas NVP-TAE684 (Alk inhibitor; **J**) and AT7867 (Akt inhibitor; **K**) induced shrinkage measured by a significant decrease in side scatter (**L**). RBCs were treated with the respective compounds for 30 min at 37°C followed by flow cytometry. All compounds were diluted in DMSO and added at 10 μM final concentration. Plots represent one of three independent measurements. Quantification of cell side scatter (SSC-A) upon DMSO, NVP-TAE684 and AT7867 treatment (**L**); results shown represent mean±S.D., *n*=3; *P<0.1, **P<0.01, unpaired *t* test was applied during the analysis.

After analysis of the induction screens, we identified 12 compounds from LOPAC inducing cell shrinkage/vesiculation and 10 compounds from kinase inhibitor set inducing cell shrinkage/vesiculation. All validated hits from LOPAC and kinase inhibitor induction screens are listed in Tables 1 and 2 respectively. In case of the LOPAC screen, the description includes relevant effects of the compounds not only provided by Sigma (denoted by a star *) but also functions described in literature. Validated hits had a median of the replicates value (Rep median score) higher than 1, meaning they caused an increase in the number of events in P2 compared with controls. Nevertheless, four LOPAC compounds were identified by analysis of RBC scatter alone and not by score value for the following reasons: palmitoyl-DL-carnitine chloride and calmidazolium chloride induced massive vesiculation in RBC leading to a concentration of all events in P3 (Figures 2A and 2B), therefore an increase in P2 could not be used as a parameter. SCH-202676 hydrobromide, on the other hand, induced vesiculation measured by an increase in P3 but no shrinkage (Figure 2C), whereas rac-2-ethoxy-3-hexadecanamido-1-propylphoshocholine's effect on RBC scatter was so strong, no events could be measured (Figure 2D). The adjusted *P*-value (Padj score) of all hits in the induction screens was ≤0.1, which was considered significant, with the exception of bromoacetyl alprenolol menthane and NNC 55–0396 from the LOPAC (Table 1) and AT7867 and NVP-TAE684 from the Kinase Inhibitor Library (Table 2). Nevertheless, these compounds were included in the validated hit list as they all had Rep median score higher than 1 (Tables 1 and 2). In addition, these compounds induced RBC shrinkage/vesiculation (Figures 4H, 4I, 5J and 5K), measured by a decrease in RBC side scatter, indicating reduced cell size (Figures 4J and 5L). The compounds from the LOPAC library inducing cell shrinkage/vesiculation could be clustered into four functional groups: PKC activity (six compounds including ET-18-OCH3 [34], calcium signalling (two compounds), GPCR signalling (three compounds) and protease activity (one compound) [35] (Figure 3A). Overall these results underscored the potential of our screening method, since the effects of PKC signalling [28], Ca^2+^ [26] and calpain [27] on RBC have been well studied. In addition, we identified GPCR signalling for the first time to be involved in RBC vesiculation. Figures 2 and 4 illustrate the effects of all LOPAC hits on RBC scatter. Compared with RBC treated with DMSO alone (Figure 4A), it is clear that all validated hits induce RBC shrinkage, measured by a significant increase in the number of events in P2 (Figures 4B–4G), with the exception of bromoacetyl alprenolol menthane and NNC 55–0396 (Figures 4H and 4I), which induced RBC shrinkage measured by a decrease in RBC side scatter (Figure 4J). Since the Kinase Inhibitor Library consisted exclusively of specific kinase inhibitors, we clustered the validated hits according to the family of kinases they target (Figure 3B). Interestingly, we discovered not only compounds targeting kinases with known functions in RBC to induce vesiculation (Table 2), but also compounds inhibiting kinases were described to be expressed in RBC, such as Alk [36] and VEGFR-2 [37]. We could confirm VEGFR-2 was found in RBC with a Western blot (result not shown). Figure 5 shows representative scatter plots of RBC treated with all validated kinase inhibitors. All induction compounds caused a significant increase in P2 compared with treatment with DMSO alone (Figures 5B–5I), with the exception of NVP-TAE684 and AT7867 (Figures 5J and 5K), which induced shrinkage measured by a significant decrease in RBC side scatter (Figure 5L). In addition, NVP-TAE684 treatment lead to vesiculation, measured by an increase in events in P3 (Figure 5J). Moreover, we generated response plots of the hits identified in the kinase inhibitor induction validation screen demonstrating how strong the effect of each compound was (Supplementary Figure). Compound concentrations (100 nM, 1 μM and 1 0μM) were plotted on the *x*-axis whereas the normalized P2 value was plotted on the *y*-axis. The response to each compound was represented by a solid line. The closer the line was to the positive control (dotted red line), the stronger the effect was. No compounds inhibiting vesiculation upon ionomycin stimulation were identified during the LOPAC validation inhibition screen and the Kinase Inhibitor Library inhibition screen.

**Table 1:** List of validated LOPAC compounds inducing RBC shrinkage and vesiculation

Rep median score	Padj score	Name	Description
1.223	0.036	ATA	Calpain inhibitor^[35]^
1.232	0.036	ET-18-OCH3 (edelfosine)	PLC inhibitor*, PKC inhibitor^[34]^
1.241	0.036	Rottlerin	PKC inhibitor*
1.277	0.036	Reactive Blue 2	P2Y receptor antagonist*
1.255	0.036	PMA	PKC activator*
1.194	0.089	Tamoxifen citrate	PKC inhibitor, oestrogen receptor antagonist*
1.088	0.102	Bromoacetyl alprenolol menthane	β-AR antagonist*
1.109	0.107	NNC 55-0396	T-type calcium channel inhibitor*
		SCH-202676 hydrobromide	GPCR modulator*
		Palmitoyl-DL-carnitine chloride	Modulator of PKC activity*
		rac-2-Ethoxy-3-hexadecanamido-	PKC inhibitor*
		1-propylphosphocholine	
		Calmidazolium chloride	CaM inhibitor*

**Table 2:** List of validated Kinase Inhibitor Library compounds inducing RBC shrinkage and vesiculation

Rep median score	Padj score	Name	Target
1.283	0.001	AP24534	pan–BCR–ABL
1.229	0.004	AS-252424	PI3kγ
1.173	0.005	Sorafenib tosylate	Raf-1,B-Raf, VEGFR-2
1.068	0.009	SNS-314 mesylate	Aurora kinases
1.122	0.017	A-769662	AMPK activator
1.17	0.034	BIRB 796	p38 MAPK
1.203	0.037	CX-4945	CK2
1.05	0.076	NVP-BSK805	Jak-2
1.027	0.107	AT7867	Akt
1.027	0.257	NVP-TAE684	ALK

### CK2 regulates RBC shrinkage via modulation of the Gardos channel

**Figure 6:**
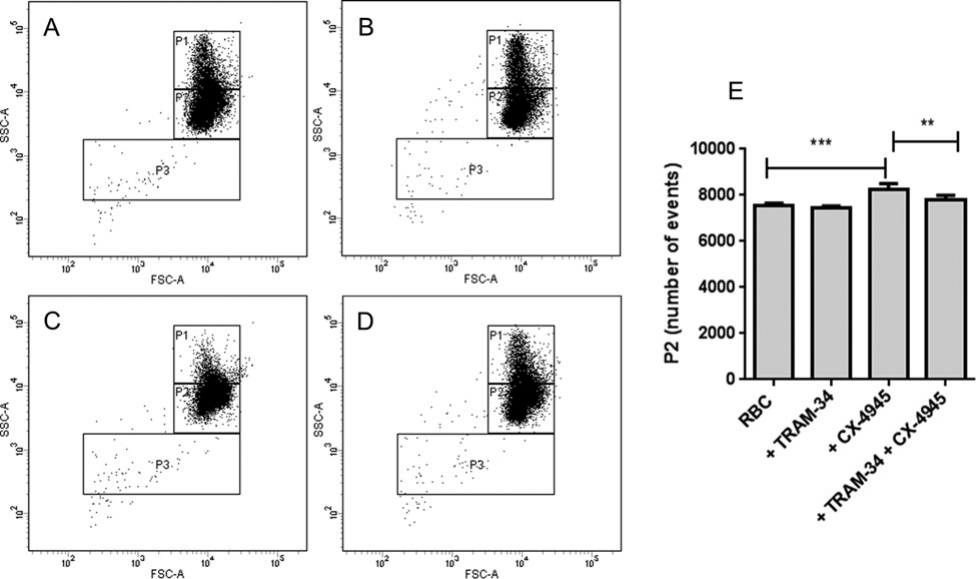
RBC shrinkage induced by CX-4945 is mediated via the Gardos channel RBCs were treated with DMSO alone as a control (**A**), 20 μM TRAM-34 (Gardos channel inhibitor; **B**), 10 μM CX-4945 (CK2 inhibitor; **C**) and TRAM-34 followed by CX-4945 (**D**). RBCs were treated with each compound for 30 min at 37°C followed by flow cytometry. CK2 inhibition lead to RBC shrinkage measured by an increase in events in P2 (**C**), which could be prevented by blocking the Gardos channel prior to the addition of CX-4945 (**D**). Plots represent one of six independent measurements. Statistical analysis of CX-4945 effect on RBC shrinkage with or without TRAM-34 treatment (**E**); results shown represent mean±S.D., *n*=6; **P<0.01, ***P<0.001, unpaired *t* test was applied during the analysis.

We discovered two compounds inhibiting CK2 (CX-4945 and AS-25245) to induce RBC shrinkage measured by an increase in the number of events in P2 (Figures 5B, 5C, 6C, and 6E). Interestingly, CX-4945 effect on RBC volume was abrogated once the Gardos channel was blocked with the specific inhibitor TRAM-34. We could clearly see that inhibition of CK2 with CX-4945 did not induce RBC shrinkage when the cells were pre-treated with TRAM-34 (Figures 6D and 6E). These results suggest that RBC shrinkage caused by CK2 inhibition is mediated via the Gardos channel. Inhibition of the channel alone did not induce any changes on RBC scatter (Figure 6B).

## DISCUSSION

In the present study, we successfully identified novel pathways involved in RBC vesiculation through screening of two libraries of pharmacological inhibitors. RBC vesiculation is not only relevant in the context of RBC clearance and repair under physiological conditions, but also in the context of blood transfusions. Vesicles that form during storage can cause adverse effects in the recipient including NO scavenging [9–11] and promotion of coagulation [6–8]. Therefore, it is imperative that we unravel the signalling cascades contributing to these processes. To induce vesiculation, we used the calcium ionophore ionomycin, which causes calcium influx and potassium efflux via the Ca^2+^-activated potassium channel (Gardos channel) which leads to RBC shrinkage and vesiculation (Figure 1B). In accordance with the evidence in literature regarding the role of Ca^2+^ in RBC vesiculation, we found two compounds related to Ca^2+^ signalling to induce vesiculation in RBC: calmidazolium chloride (CaM antagonist) and NNC 55–0396 (T-type Ca^2+^ channel blocker). The calcium sensor CaM has been implicated in the regulation of RBC shape and membrane stability by modulating cytoskeletal interactions [38]. The mechanism by which calmidazolium chloride induces vesiculation is probably via inhibition of the Ca^2+^ ATPase pump which is regulated by CaM. Suppression of Ca^2+^ ATPase activity leads to ATP depletion and accumulation of calcium inside the cell [39], similar to the events observed during ionomycin treatment. Furthermore, calmidazolium chloride has been shown to inhibit phosphodiestarases (PDEs) in various cell types, which could elevate cAMP/cGMP levels inside the cell, thereby potentially influencing RBC vesiculation as well [40]. Moreover, as a Ca^2+^-activated potassium channel, the Gardos channel has a CaM-binding domain which facilitates the binding of CaM to the channel [41]. Nevertheless, a previous study has indicated that CaM antagonists including calmidazolium do not inhibit Gardos channel's activity [42], which implies that the massive vesiculation observed upon calmidazolium treatment (Figure 2B) does not involve direct effects on the Gardos channel. Furthermore, calcium flux in RBC can activate the non-lysosomal protease calpain μ-type [43]. Aurintricarboxylic acid (ATA) is a general apoptosis inhibitor capable of inhibiting DNA topoisomerase II [44] and calpain [35]. Calpain is known to cleave the Ca^2+^ ATPase pump [45], PKC [46], spectrin, ankyrin and protein 4.1 [47] and is considered a main regulator of RBC physiology and deformability [27]. It was also demonstrated that calpain inhibition ameliorates the red cell phenotype in a SCD mouse model via impairment of Gardos channel activity [48]. Furthermore, calpain activation has been directly linked to microparticle formation in platelets [49]. All these data suggest that inhibition of calpain activity would rather prevent vesiculation than trigger it, nevertheless our results demonstrate that calpain inhibition with ATA induces RBC shrinkage (Figure 4B) and 100% PS exposure (result not shown). We propose that inhibition of calpain activity has a complex role in RBC, including the induction of shrinkage and possibly the release of nanovesicles under normal conditions (Figure 4B).

In addition, we identified six inducers of shrinkage and vesiculation from the LOPAC library related to PKC activity. These include the inhibitors rottlerin, tamoxifen citrate, rac-2-ethoxy-3-hexadecanamido-1-propylphosphocholine, ET-18-OCH3, the modulator palmitoyl-DL-carnitine chloride and interestingly the activator PMA. There are five PKC isoforms expressed in RBC described in literature: classical α, atypical ζ [50], conventional β [51], atypical ι and PKC μ [52]. From these, PKCα [52] and PKC*β* [51] translocate to the membrane upon stimulation with PMA which suggests that RBC vesiculation is promoted either via activation of PKCα and PKCβ or via inhibition of the other isoforms present in RBC. PKC has a myriad of functions in RBC related to shrinkage [28], PS exposure [53] and Ca^2+^ flux [54]. Since all of these phenomena are related to RBC vesiculation, it was not surprising that we identified six compounds exerting effects on PKC to be involved in vesiculation (Table 1). In addition, PKC down modulates Gardos channel activity [55], which hints to the possibility of K^+^ efflux stimulation upon PKC inhibition, ultimately resulting in vesiculation. Furthermore, as inducers of vesiculation we identified nine kinase inhibitors and one kinase activator from the Kinase Inhibitor Library which we divided in four functional groups (Figure 3B) depending on the family of kinases they target (Table 2). A-769662, the only activator, targets AMPK (AMP-activated kinase) [56], an energy sensor and regulator of metabolism, expressed in skeletal muscle, liver, pancreas and adipose tissue [57]. AMPK is activated by AMP, hence its name AMP-activated kinase, as well as by Ca^2+^/CaM-dependent protein kinase kinase β and liver kinase B1 [58]. AMPK is also expressed in RBC and has been previously proposed to have a role in RBC survival. Ampk^−/−^ mice present with anaemia and splenomegaly and are cleared faster from circulation [59], which suggests that AMPK might be involved in RBC vesiculation as well. Furthermore, Sid et al. [60] discovered that adding A-769662 to erythrocytes not only activates AMPK but also leads to the phosphorylation of the downstream target Na-K-Cl co-transporter (NKCC1) which might result in cell shrinkage. BIRB 796, also known as doramapimod, is a highly selective p38 mitogen-activated protein kinase (MAPK)α inhibitor [61], also shown to inhibit c-Jun N-terminal kinase 2 (Jnk-2) at 10 μM. p38 MAPK is involved in various cellular processes including regulation of cell volume by activating Na^+^-H^+^ exchanger [62]. Gatidis et al. [63] have proposed that p38 MAPK plays a role in RBC survival. Using the inhibitors SB203580 and p38 MAPK inhibitor III they showed that inhibition of p38 kinase leads to reduced PS exposure upon hyperosmotic shock or ionomycin stimulation [63]. Other studies employing pan p38 inhibitors demonstrate that p38 MAPK inhibition induces a transient delay in murine erythropoiesis [64]. *In vivo* experiments with p38 MAPK knockout mice show that mice die *in utero* or survive owing to severe anaemia due to defective erythropoiesis resulting from reduced erythropoietin (Epo) levels [65]. Moreover, inhibition of p38 MAPK abrogates erythropoiesis in human primary erythroblast cultures [66]. We demonstrate *in vitro* that p38 MAPK might influence mature RBC signalling as well by mediating RBC shrinkage (Figure 5G). Another compound validated in our induction screen, previously used in studies with RBC, is sorafenib tosylate (Bay 43–9006). Sorafenib is a marketed drug (under the name of Nexavar) approved for use against renal cell carcinoma [67], differentiated thyroid carcinoma [68] and hepatocellular carcinoma [69]. It inhibits several receptor tyrosine kinases (RTKs), such as Raf-1, B-Raf and, to a lesser extent, VEGFR-2, mPDGFRβ (m-platelet-derived growth factor receptor β) and PDGFRβ kinases [70]. As side effects of the drug might include anaemia, Lupescu et al. [71] already demonstrated that treatment of RBC with sorafenib induced shrinkage and PS exposure in RBC. Our findings further confirm that sorafenib influences RBC volume and induces vesiculation (Figure 5E), which might contribute to the anaemia observed in these patients. Furthermore, it has been reported that Raf-1 deficient embryos are anaemic and die *in utero* [72]. Thus, Raf-1 has also been described to play a role in erythropoiesis, by repressing caspase activation, hence antagonizing erythroid differentiation [73]. In addition, Raf-1 is necessary to activate MEK–ERK pathway in erythropoiesis leading to a positive feedback loop maintaining Raf-1 expression throughout the undifferentiated state [74]. Raf-1 function has not been described in mature RBC to date, thus it is intriguing to explore further how this kinase is involved in RBC signalling. Moreover, the PI3K–Akt pathway is a well-studied signalling cascade related to cell growth and survival [75]. Both kinase families and their subclasses are expressed in RBC [76]. We identified two inhibitors from this pathway to induce shrinkage in RBC: AS-252424 (Figure 5C) and AT7867 (Figure 5K). AS-252424 is a PI3K inhibitor with 30-fold selectivity for PI3Kγ over PI3Kα [77]. The Akt inhibitor AT7867 is equally potent in inhibiting all three Akt isoforms; nevertheless it targets PKA as well [78]. The PI3K–Akt pathway has been demonstrated previously to play a role in RBC deformability as Suhr et al. [76] showed that the PI3K inhibitor wortmannin reduced RBC deformability *in vitro*. Interestingly, we observed that wortmannin induced RBC vesiculation as well (result not shown), which is in accordance with the data we obtained from the kinase inhibitor screen. We speculate that the PI3K–Akt pathway is also involved in RBC shrinkage and vesiculation.

**Figure 7:**
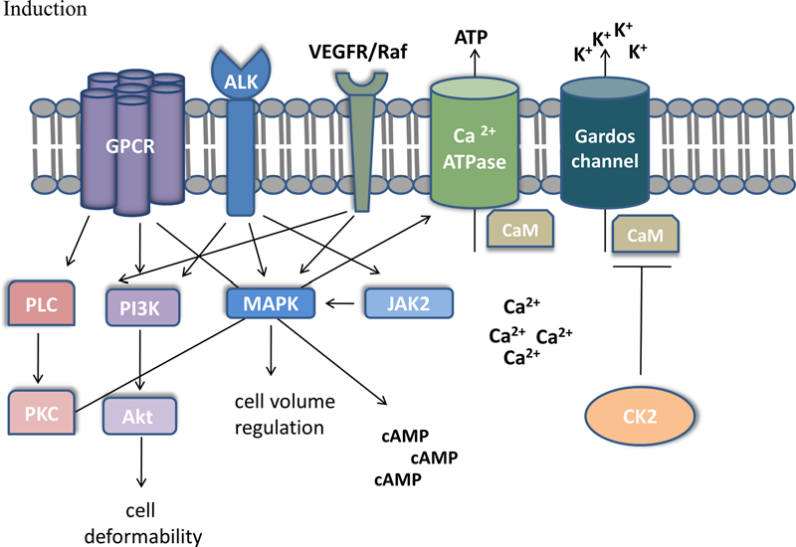
Proposed model of signalling pathways involved in RBC shrinkage and vesiculation Inhibition of the depicted kinases induces shrinkage and vesiculation in RBC. CaM antagonism causes ATP depletion and Ca^2+^ accumulation inside the cell. CK2 inhibition leads to down-modulation of CaM, which in turn activates the Gardos channel leading to K^+^ efflux and cell shrinkage. Inhibition of GPCR signalling (e.g. P2Y, β-AR) leads to cAMP increase inside the cell and ATP depletion.

To our knowledge, a T-type calcium channel, a low voltage channel, has not been described in RBC; however, there are reports of non-selective voltage activated channels in RBC [79] which have been suggested to play a role in increased pathological cation leaks in RBC [80]. Interestingly, our data suggest that T-type calcium channel activity might be related to RBC shrinkage (Figure 4I). Moreover, three compounds from the LOPAC library that we identified as vesiculation inducers are known GPCR antagonists. These are: reactive blue 2 (Basilen blue E-3G, a P2Y receptor antagonist), bromoacetyl alprenolol menthane (a β-AR antagonist; β-blocker) and SCH-202676 hydrobromide, which can act as GPCR agonist as well, since it is described as a general GPCR allosteric modulator. There is evidence in literature of GPCR signalling in RBC [81–83], even though no link to vesiculation has been established yet. P2Y receptors are purinergic GPCR activated by ATP, UDP, ADP, UTP and UDP glucose with various physiological functions including regulation of vascular tone, release of endothelial factors and platelet aggregation [84]. Blood cells express P2Y receptors from different families on their surface, whereas RBC are only known to express P2Y_1_ [85,86] and P2Y_13_ [87]. Interestingly, P2Y_13_ activation by ADP derived from ATP decreases cAMP levels in RBC and prevents ATP release. Furthermore, P2Y_13_ receptor antagonists stimulate cAMP generation and ATP release from RBC [87]. Our data suggest that treating RBC with a P2Y receptor antagonist can ultimately lead to considerable RBC shrinkage (Figure 4D), possibly due to cAMP signalling and ATP depletion [87]. Moreover, the effects of β-blockers on RBC have been intriguing scientists for a long time. There are reports from the 1970s stating that β-AR antagonists induce RBC K^+^ release [88] but the mechanisms underlying this phenomenon are still unknown. Several groups have suggested that catecholamines, such as epinephrine, have a positive effect on RBC deformability [89] and filterability [90] via a cAMP-dependent pathway as ATP-depleted RBC were unable to respond to epinephrine [91]. Our results suggest that β-blockers not only reduce RBC deformability [89], but also induce RBC shrinkage (Figure 4H). Furthermore, we discovered several novel kinases to play a role in RBC vesiculation. To our surprise, we identified the Aurora kinase pan inhibitor SNS-314 mesylate [92] as inducer of RBC vesiculation (Figure 5H). Even though Aurora kinases A and B are highly expressed in transferrin receptor (CD71^+^) early erythroid cells as shown in BioGPS gene annotation portal [93], we did not find any evidence that these kinases are expressed in mature RBC after performing a Western blot (result not shown). Therefore, it is possible that the effect of SNS-314 mesylate observed in RBC is due to an off-target compound effect. For example, SNS-314 has also been shown to inhibit Raf-1 and several RTKs such as high affinity nerve growth factor receptors (TrkA, tropomyosin receptor kinase A and TrkB, tropomyosin receptor kinase B), VEGFR-3, colony stimulating factor 1 receptor, tyrosine-protein kinase receptor UFO (Axl) and Discoidin domain receptor 2 kinase [92]. Interestingly, none of these kinases has been described to have a function in RBC. It is appealing to further investigate what the precise role of these kinases is in RBC signalling and vesiculation. Moreover, the two non-receptor tyrosine kinase (nRTK) inhibitors we discovered to induce RBC vesiculation were NVP-BSK805 (Figure 5D), targeting Jak2 and AP24534 (Ponatinib; Figure 5F), a pan BCR-ABL (breakpoint cluster region protein–Abelson murine leukaemia viral oncogene homologue 1) inhibitor. NVP-BSK805 is a Jak2 kinase inhibitor with effects towards Jak1, Jak3 and Tyk2 kinase as well [94]. Jak2 mediates Epo signalling and is essential during erythropoiesis [95]; however, even though Jak2 is expressed in mature RBC [96], which we could also demonstrate with a western blot (result not shown), its function in RBC is unknown. Interestingly, our results suggest that Jak2 might modulate RBC vesiculation. AP24534 (Ponatinib) is a pan BCR–ABL, including BCR–ABLT315I, inhibitor used in the clinic to treat chronic myeloid leukaemia; however, AP24534 exhibits inhibitory activity towards VEGFR-2, FGFR-1 (fibroblast growth factor receptor-1), scr and Lyn (protein tyrosine kinase Lyn) kinases as well [97]. Reports demonstrate that Lyn is essential for erythropoiesis and Epo-receptor signalling [98]. Furthermore Lyn directly phosphorylates Band-3 [99], thus regulating RBC shape and cytoskeletal rearrangement in healthy RBC. Moreover, Lyn is involved in the pathology of acanthocytosis [100]; nevertheless its role in RBC vesiculation has not been addressed. Another target of AP24534 is the nRTK VEGFR-2. Expression of VEGFR-2 in RBC is debatable. Sachanonta et al. [37] have shown that malaria-infected RBC and RBC stain positive for VEGFR-2, even though the authors speculated that the detected expression might be due to passive uptake of the receptor by RBC from serum. We have confirmed that VEGFR-2 is present in RBC by a Western blot (result not shown) and our data suggest that VEGFR-2 inhibition regulates RBC volume. VEGFR-2 activates PI3K–Akt pathway and PLC (phospholipase C)–PKC pathway [101], thus it is tempting to speculate that VEGFR-2 is involved in vesiculation as well since downstream targets of VEGFR-2 are known to have various roles in RBC signalling. The only RTK inhibitor we found to induce RBC vesiculation was NVP-TAE684, an Alk RTK inhibitor [102]. Alk kinase is upstream of various signalling pathways such as the MAPK–ERK, the Jak–STAT and the PI3K–Akt pathway [103]. Alk kinase has been described to be expressed in RBC only recently [36] and since our screen results showed that Alk inhibition induced RBC vesiculation (Figure 5J), it is possible that this kinase is involved in RBC signalling as well. In addition, we discovered two inhibitors of CK2: CX-4945 (Silmitasertib), a specific inhibitor [104] and AS-252424, inhibitor at 10 μM [77] that induced RBC shrinkage (Figures 5B an 5C). CK2 is highly expressed in RBC and has been shown to play a role in immune adherence clearance [105] and to mediate membrane deformability upon complement receptor 1 ligation [106]. Interestingly, in neurons CK2 binds to the Ca^2+^-activated potassium channel SK2 (small conductance calcium-activated potassium channel 2), thus directly regulating its function [107]. Since SK2 requires CaM signalling for proper functioning, CK2 phosphorylation of CaM abrogates SK2 channel's sensitivity to calcium leading to SK2 inactivation [107]. Interestingly, the Gardos channel, as a small conductance calcium-activated potassium channel, shares homology with the other SK channels [41]. Involvement of CK2 in the activity of the Gardos channel in RBC has not been demonstrated. We suggest that RBC shrinkage induced by CK2 inhibition is mediated via the Gardos channel since blocking of the channel with the specific inhibitor TRAM-34 prevents shrinkage induced by CX-4945 (Figure 6). We speculate that, similar to what is observed in neurons, CK2 might be co-assembling with the Gardos channel, modulating its function [107]. Inhibition of CK2 would prevent CaM phosphorylation, which could lead to constitutive activation of the potassium channel due to increased Ca^2+^ sensitivity [108], resulting in cell shrinkage. Certainly, a demonstration of the direct interaction between CK2 and the Gardos channel is necessary to further confirm their concerted role in RBC vesiculation. Lastly, we did not identify any compounds inhibiting vesiculation upon ionomycin stimulation. One reason for the lack of hits in the inhibition screens could be the challenging task to abrogate the strong effect of RBC vesiculation induced by ionomycin. In conclusion, we discovered several novel signalling cascades to be involved in RBC vesiculation, including GPCR signalling, the PI3K–Akt pathway, the Raf–MEK–ERK pathway, and the Jak–STAT pathway. Moreover, we suggest for the first time a role of CK2, Alk kinase and VEGFR-2 in RBC shrinkage and vesiculation (Figure 7). We cannot exclude the possibility of redundancy in some of these pathways and more research is needed to elucidate the exact functions of these cascades in RBC vesiculation and the storage lesion.
